# Complete Genome Sequence of a Recombinant Porcine Reproductive and Respiratory Syndrome Virus Strain from Two Genotype 1 Modified Live Virus Vaccine Strains

**DOI:** 10.1128/genomeA.00454-17

**Published:** 2017-06-01

**Authors:** Patricia Renson, Fabrice Touzain, Arnaud Lebret, Mireille Le Dimna, Hélène Quenault, Valérie Normand, Jean-Baptiste Claude, Floriane Pez, Nicolas Rose, Yannick Blanchard, Olivier Bourry

**Affiliations:** a ANSES, Ploufragan-Plouzané Laboratory, Swine Virology and Immunology Unit, Zoopôle, Ploufragan, France; b ANSES, Ploufragan-Plouzané Laboratory, Swine Epidemiology and Welfare Unit, Zoopôle, Ploufragan, France; c ANSES, Ploufragan-Plouzané Laboratory, Viral Genetics and Biosecurity Unit, Zoopôle, Ploufragan, France; d Université Bretagne Loire, Rennes, France; e Biosellal, Dardilly, France; f UGPVB, Rennes, France; g Porc. Spective, Groupe Vétérinaire Chêne Vert Conseil, Noyal-Pontivy, France

## Abstract

This paper provides information on the complete genome sequence of a porcine reproductive and respiratory syndrome virus (PRRSV) strain isolated on a French pig farm which was identified as a recombinant strain from two commercial modified live virus vaccine strains of genotype 1 (VP-046BIS and DV strains).

## GENOME ANNOUNCEMENT

Porcine reproductive and respiratory syndrome (PRRS) is considered the most costly disease for the pig industry worldwide (1). The disease is characterized by reproductive failure in sows and respiratory disorders and growth retardation in growing pigs (2). The causative agent is an enveloped virus with a 15-kb positive-polarity single-stranded RNA genome, and it is a member of the *Arteriviridae* family that encodes 10 open reading frames (ORFs). Strains of PRRS virus (PRRSV) cluster into either European genotype 1 or North American genotype 2 (3).

To control PRRSV, modified live virus (MLV) vaccines of genotypes 1 (MLV1) and 2 (MLV2) are widely used. In France, only genotype 1 PRRSV strains have been documented, and so only MLV1 vaccines are licensed. The PRRS-FR-2014-56-11-1 strain was isolated in pulmonary macrophages from healthy piglet serum samples collected in December 2014 in a French pig farm following a PRRS stabilization program. The farm had a history of PRRSV infection, and PRRS vaccination was implemented using first the Unistrain vaccine (VP-046BIS strain; Hipra) and then the Porcilis vaccine (DV strain; MSD). At the end of 2013, a batch of almost 500 piglets was unintentionally vaccinated with both strains a few weeks apart. The full-genome sequence of the PRRS-FR-2014-56-11-1 strain was obtained using next-generation sequencing (NGS) at the ANSES NGS platform and at the Biosellal laboratory.

At ANSES, the strain was sequenced with a proton sequencer. Raw reads were downsampled to get an estimated PRRSV coverage depth of 80×. KmerGenie 1.5658 (4) and the Mira 4.0rc1 assembler (5) were then provided with reads. Only viral contigs of the *de novo* assembly were retained. Manual corrections were made based on Tmap (Torrent Suite 4.0.2) alignment of reads cleaned by Trimmomatic 0.32 (6) on the *de novo* assembly. At Biosellal, the strain was sequenced with an Illumina MiSeq platform. The paired reads that were obtained were trimmed and mapped against the DV strain genome (GenBank accession number KJ127878) with CLC Genomics Workbench 8.5.1 (Qiagen). The final coverage depth was above 1,000×.

The full-genome sequences from ANSES and Biosellal were 100% identical. The full-genome sequence of PRRS-FR-2014-56-11-1 showed 97.5% (nucleotide) identity with VP-046BIS and 94.5% with DV. Using the SimPlot program (7), the recombinant origin of the PRRS-FR-2014-56-11-1 strain was assumed. The RDP4 software (8) made it possible to identify three recombination events in VP-046BIS (major parent) and DV (minor parent) at nucleotide positions 500 to 1370, 3646 to 4272, and 4972 to 8430, all located in ORF1 encoding the viral RNA replicase.

To date, recombinant strains have been identified either between PRRSV field strains (9) or between field strains and MLV vaccine strains (10). Here, we report the first mosaic isolate combining two MLV vaccine strains.

The question naturally raised by this finding is whether this strain could be virulent. To answer this question, an *in vivo* evaluation should be conducted rapidly. Nevertheless, considering that (i) the PRRS-FR-2014-56-11-1 strain is a combination of two attenuated strains and (ii) no clinical signs evocative of PRRS were detected in the farm, the virulence level is expected to be fairly low.

### Accession number(s).

The PRRS-FR-2014-56-11-1 genome sequence has been deposited in GenBank under the accession no. KY767026.
